# Crystal Structure of a Complex of NOD1 CARD and Ubiquitin

**DOI:** 10.1371/journal.pone.0104017

**Published:** 2014-08-15

**Authors:** Aaron M. Ver Heul, Lokesh Gakhar, Robert C. Piper, Ramaswamy Subramanian

**Affiliations:** 1 Molecular Physiology and Biophysics, University of Iowa, Iowa City, Iowa, United States of America; 2 Department of Biochemistry, University of Iowa, Iowa City, Iowa, United States of America; 3 Carver College of Medicine Protein Crystallography Facility, Iowa City, Iowa, United States of America; 4 Institute for Stem Cell Biology and Regenerative Medicine, Bangalore, India; Indian Institute of Science, India

## Abstract

The Caspase Recruitment Domain (CARD) from the innate immune receptor NOD1 was crystallized with Ubiquitin (Ub). NOD1 CARD was present as a helix-swapped homodimer similar to other structures of NOD1 CARD, and Ub monomers formed a homodimer similar in conformation to Lys48-linked di-Ub. The interaction between NOD1 CARD and Ub in the crystal was mediated by novel binding sites on each molecule. Comparisons of these sites to previously identified interaction surfaces on both molecules were made along with discussion of their potential functional significance.

## Introduction

NOD1 is an innate immune receptor that recognizes γ-D-glutamyl-meso-diaminopimelic acid (iE-DAP), a dipeptide present in peptidoglycan of bacteria, and activates inflammation and autophagy in response to bacterial infection [1], [2]. Loss of NOD1 function increases susceptibility to severe systemic infections and is associated with a variety of inflammatory and autoimmune syndromes in humans [3]–[8]. NOD1 is composed of three domains: an N-terminal Caspase Recruitment Domain (CARD), a central nucleotide-binding oligomerization domain and a C-terminal ligand-binding domain [9]. Upon ligand binding, NOD1 oligomerizes into a signaling scaffold, which in turn recruits downstream effector molecules including kinases and ubiquitin ligases [10].

The N-terminal CARD of NOD1 has been shown to coordinate interactions with its various effectors. CARDs are part of a larger family of protein interaction motifs known as Death Domains (DD). In addition to CARDs, the DD superfamily includes Death Domains, Death Effector Domains and Pyrin Domains. These domains are typically around 100 amino acid residues in length and share a common six-helical bundle structural fold. They are involved in almost all aspects of immune and apoptotic signaling, and are found on various receptors, such as NOD1, as well as downstream signaling molecules such as RIP2, the effector kinase for NOD1 [11]. The majority of these domains have yet to be characterized structurally, and there are even fewer data describing how DDs complex with effector proteins. Like other DD subfamilies, CARDs were thought to engage almost exclusively in homotypic interactions with other CARDs [11]. Recently, however, Ub has been found to bind the CARDs of the innate immune receptors NOD1, NOD2, RIG-1 and MDA5 and regulate their activity [12]–[14].

Ub functions when covalently attached to substrate proteins as either a single mono-Ub or as a poly-Ub chain where distal Ubs are linked by their C-termini to one of seven lysine residue sidechains or the N-terminus of a proximal Ub moiety. Lys48-linked poly-Ub targets substrates to the proteasome for degradation, and this process plays an important role in immune signaling through destruction of IκB, which is required to release the cytosol-bound NF-κB transcription factor to activate genes involved in the immune response [15]. In contrast, linear Met1-linked or Lys63-linked poly-Ub chains form platforms assembling Ub-binding kinases and other effectors that activate signaling pathways (reviewed by [16]–[19]). In some pathways, Lys63-linked poly-Ub chains alone are sufficient to nucleate effector complexes and activate signaling [20].

The interactions between Ub and its effectors are complex and only a relatively small proportion of them have been characterized structurally. Here we report the crystal structure of a complex of NOD1 CARD and Ub. NOD1 CARD is present as a domain-swapped homodimer, similar to previously reported structures [21], [22], while two Ub monomers interact in a conformation similar to that of Lys48-linked di-Ub [23]. We discuss the implications of this structure with regard to how NOD1 and other Ub-binding proteins may recognize Ub *in vivo*.

## Results

### Both subunits of the NOD1 CARD dimer interact with a single Ub moiety

**Figure 1:**
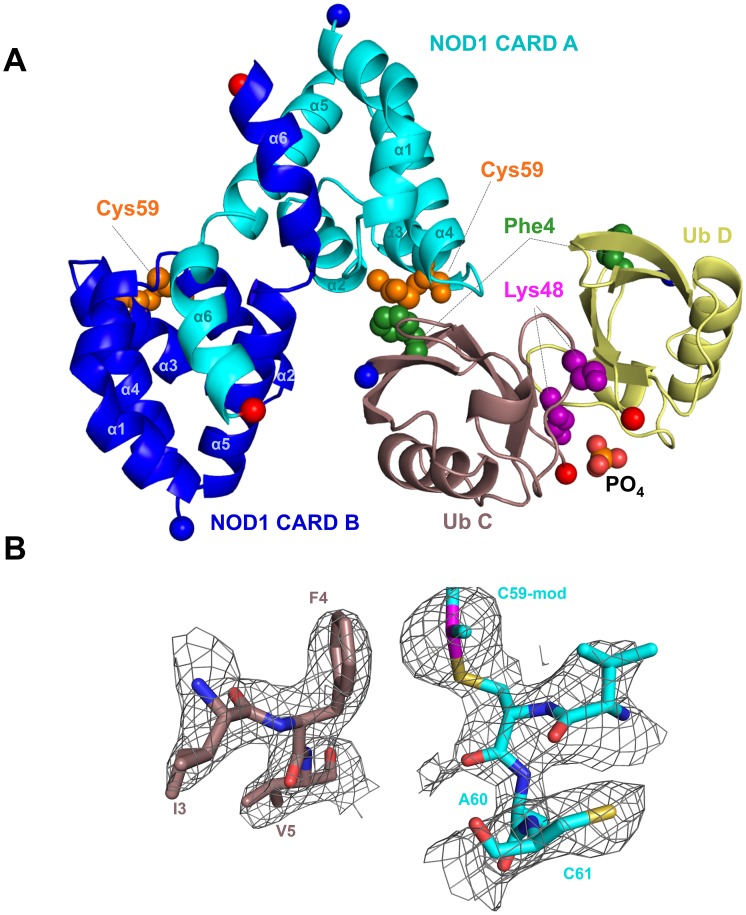
Cartoon view of the NOD1 CARD-Ub complex. **A**) NOD1 CARD and Ub crystallized as a dimer of dimers. NOD1 CARD A (cyan) and Ub C (mauve taupe) are in the asymmetric unit. Symmetry-related subunits NOD1 CARD B (blue) and Ub D (yellow) complete the full complex. The NOD1 CARD homodimer demonstrates swapping of the sixth helices, similar to other reported NOD1 CARD dimers (2NSN and 2NZ7). The ubiquitin dimer chelates a phosphate anion (shown as spheres), similar to chelation of sulfate seen in another structure of Lys48-linked tetra-Ub (2O6V). Phe4 of Ub (green spheres) and the modified Cys59 of NOD1 CARD (orange spheres) are key residues mediating the NOD1 CARD-Ub interaction. The Lys48 residues of both Ub molecules are shown as magenta spheres. The N- and C- termini are highlighted as blue and red spheres, respectively. **B**) Close-up view of the dimethyl-arsenic adduct on NOD1 CARD. The Cys59 sidechain of NOD1 CARD (cyan) is shown in isolation. The adduct is within ∼3.5 Å of the aromatic Phe4 sidechain on Ub (mauve taupe), also shown in isolation. Electron density contoured at 2.0 sigma and carved at 2.3 Å shows the Cys59 sidechain is modified (left), while the nearby solvent-exposed Cys61 is not.

The asymmetric unit of the crystal structure contains one NOD1 CARD and one Ub, while symmetry-related molecules reveal that both NOD1 CARD and Ub form homodimers (Figure 1A). As observed in two previous crystal structures, NOD1 CARD formed a homodimer that involved swapping of the sixth helix of each subunit [21], [22]. Consistent with the previous structure determined by Coussens *et al*, the two Cys39 sidechains at the dimer interface in our structure remained reduced (Figure 1B). Superposition of the NOD1 CARD dimer determined in this structure with those previously determined shows close similarity with RMSD values of 1.21 Å and 1.79 Å over the Cα chain for 2NSN [22] and 2NZ7 [21], respectively.

During refinement, we noticed a large volume of electron density immediately adjacent to the sidechain of Cys59 on NOD1 CARD that could not be accounted for by a sulfhydryl alone (Figure 1D). Given that NOD1 CARD was maintained in buffer containing 5 mM DTT from its initial purification, the likelihood of oxidative modification of the sulfhydryl of Cys59 was quite low. DTT has been observed to form adducts with Cys sidechains [24], but in this case the density we observed was not sufficient to account for that particular addition. The buffer in the crystallization condition contained cacodylate, which has been shown to form arsenic (As) adducts with proteins [25]–[27]. In the presence of DTT, pentavalent cacodylate can be reduced to a highly reactive trivalent dimethyl-As-DTT intermediate. This intermediate reacts with surface exposed Cys residues on proteins, leading to addition of dimethyl-arsenic moieties to the sidechain sulfhydryls [28], [29]. Thus, the components of the crystallization buffer strongly suggested the possibility of chemical modification of NOD1 CARD with dimethyl-As. Subsequent refinement of the model with a dimethyl-As moiety on Cys59 resulted in a good fit with the observed electron density (Figure 1B), and we found no evidence that either of the other two cysteine sidechains (Cys39 or Cys61) were similarly modified.

### Ub forms a homodimer similar in conformation to Lys48-linked Di-Ub

**Figure 2:**
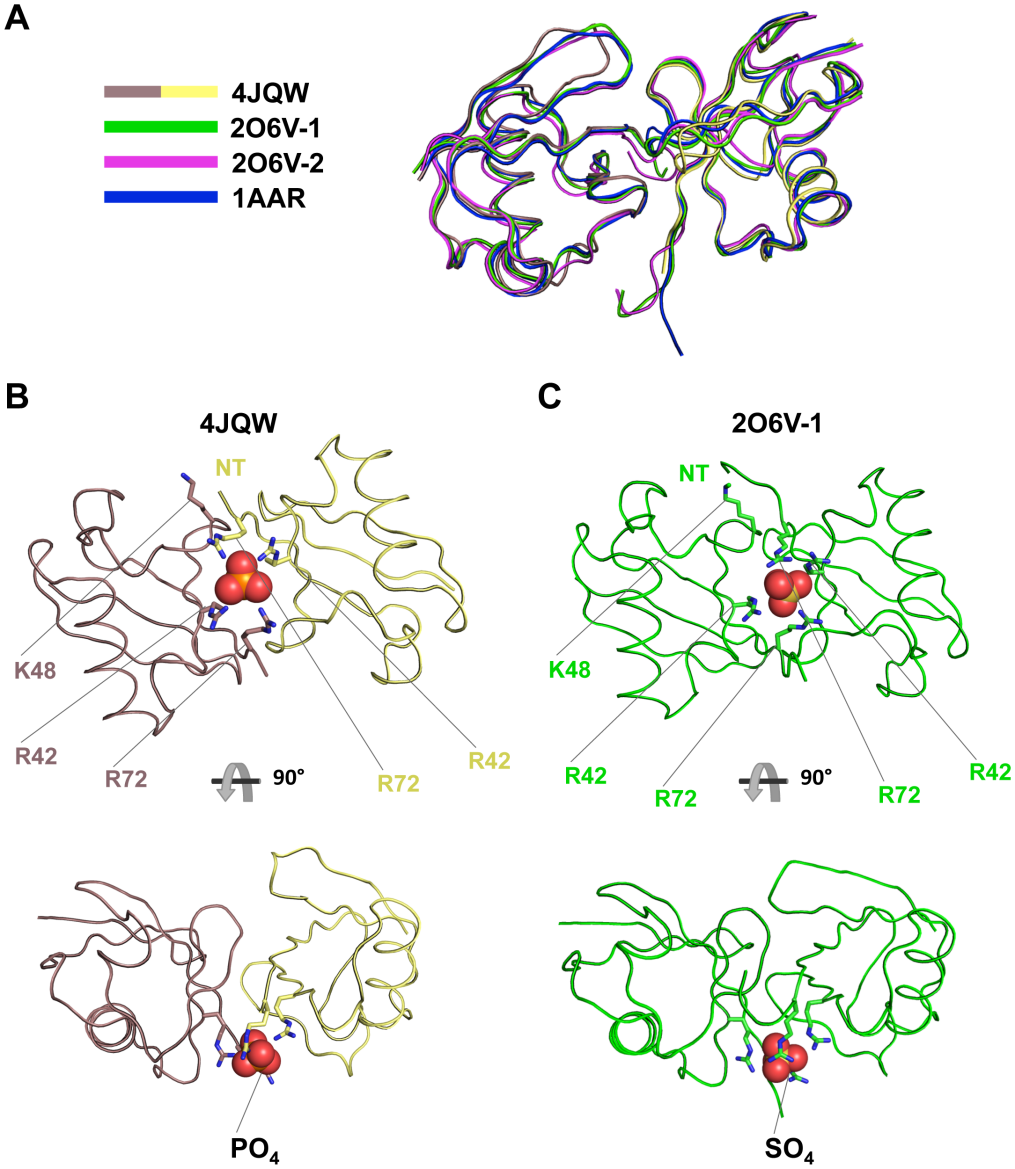
Mono-Ub in our structure formed dimers similar in conformation to other structures of K48-linked di-Ub. **A**) Ribbon representation of an alignment of K48-linked diubiquitin structures. The proximal di-Ub (2O6V-1, green) and distal di-Ub (2O6V-2, magenta) from tetra-Ub and di-Ub from 1AAR (blue) were aligned to the Ub dimer structure reported in this study (Ub C, mauve taupe and Ub D, yellow). Main chain RMSDs for this alignment were 1.309, 0.892 and 0.737 Å, respectively. **B**) Loop representation of the Ub dimer from our structure shown chelating a phosphate anion. This chelation is mediated mainly by Arg42 and Arg72 from each subunit. **C**) Loop representation of the proximal di-Ub moiety from K48-linked tetra-Ub (2O6V-1). In this case, Arg42 and Arg72 mediate chelation of a sulfate anion. In both structures, the Arg residues, which are located at the periphery of the hydrophobic pocket and often participate in interactions with UBDs, are engaged instead through these chelation interactions. This may serve as a mechanism by which UBDs discriminate between K48-linked poly-Ub and other forms of poly-Ub. K48 of the distal Ub moiety and the N-terminus (NT) of the proximal Ub moiety of each dimer are shown, demonstrating the close proximity in the dimer relative to the covalently linked chain.

Two Ubs were found in the unit cell as a homodimer in a conformation nearly identical to the structure of covalently linked Lys48 poly-Ub [23] despite the lack of any covalent bonds enforcing a particular conformation (Figure 2A). Previous studies have shown that pH can be a critical factor in determining which conformations Lys48-linked poly-Ub chains adopt, with acidic conditions favoring an open state and more neutral environments favoring a closed state, characterized by the hydrophobic patch of each Ub (comprised of Ile44, Val70 and Leu8) facing each other [23], [30]. Additionally, the Ub dimer in our structure demonstrates the chelation of a multivalent anion by the sidechains of Arg42 and Arg72 in a fashion also observed for Lys48 linked poly-Ub [23] (Figure 2B,C). Since the proportion of basic residues to acidic ones in the interface is relatively high, chelation of a large anion through dimerization may serve to mitigate this electrostatic disparity. At lower pH, protonation of the few acidic residues, as well as anionic ligands, would be expected to disrupt this chelation through charge repulsion and favor an open state of the linked Ub moieties, explaining the pH-dependence of the conformational change.

Our crystals formed at a pH of ∼8.6, significantly higher than the neutral pH values previously shown to favor inter-Ub interactions relative to acidic environments for Lys48-linked chains. Nonetheless, this closed conformation of Ub has not been observed in the absence of conformational constraint imposed by Lys48-Gly76 covalent bonds between Ub subunits, and it was a remarkable finding in our crystal structure. A recent non-covalent dimeric structure of Ub has been described which chelates cadmium [31]. However, the Ub homodimer in that structure does not match any other known poly-Ub conformation, and the effects of cadmium chelation may explain this idiosyncrasy.

### NOD1 CARD binds a unique interface on Ub

**Figure 3:**
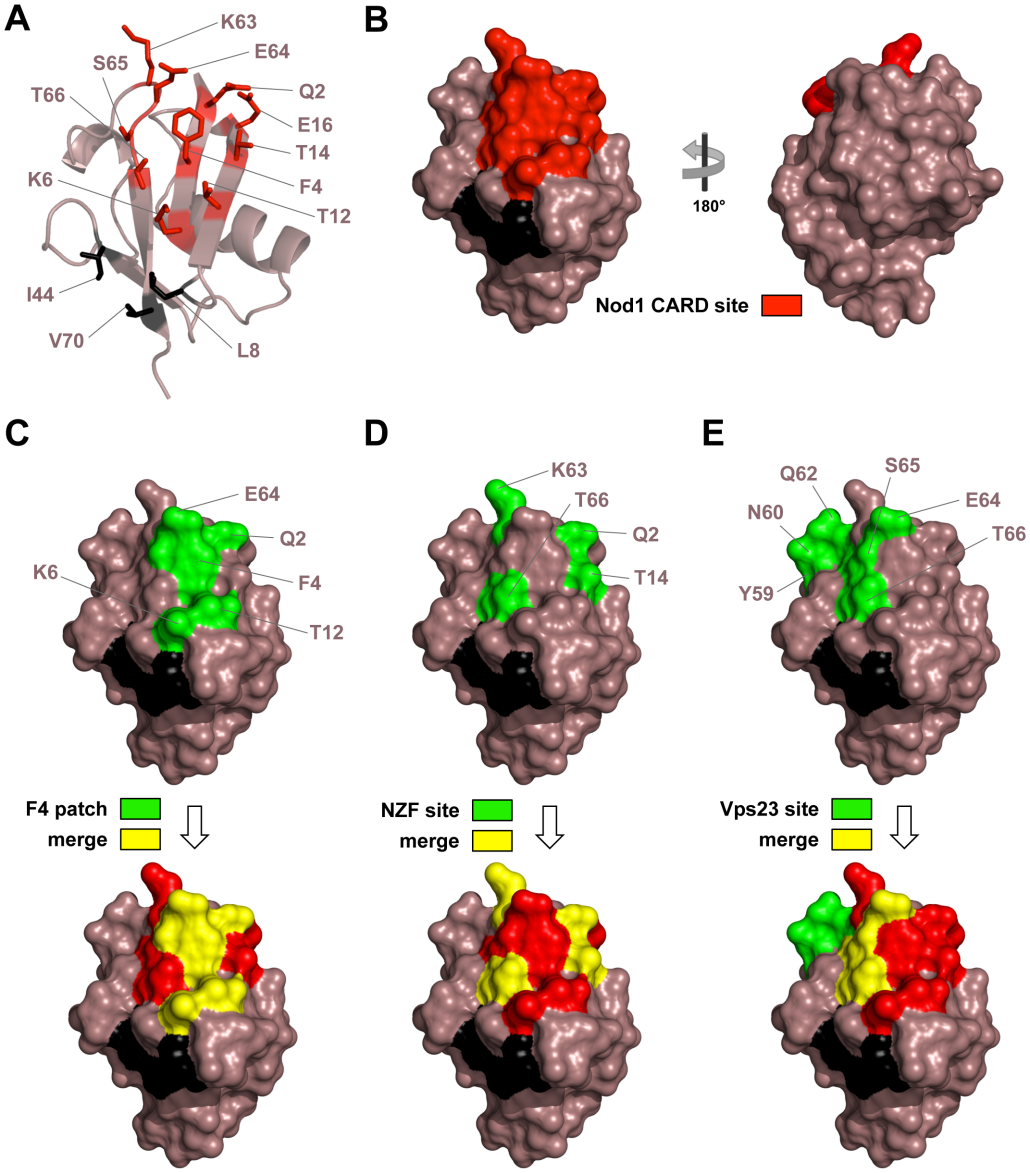
Identified UBD binding sites in Ub. Ub is colored in mauve taupe. **A**) Cartoon representation of Ub. Residues mediating interaction with NOD1 CARD are shown as sticks and are colored red. The canonical hydrophobic pocket residues are shown as sticks and colored black. **B**) Surface views of the NOD1 CARD interaction site on Ub. **C**) Surface views of the Phe4 hydrophobic patch (F4 patch) on Ub alone (green) and merged with the NOD1 CARD site (yellow). **D**) Surface views of one site on Ub recognized by the NZF domain of HOIL-1L alone (green) and merged with the NOD1 CARD site (yellow). This domain specifically recognizes linear chains of Ub by binding to the hydrophobic pocket of the distal Ub and to an area surrounding Phe4 of the proximal Ub of a linear di-Ub moiety. Residues on the proximal Ub interacting within 3.6 Å of the NZF domain in a crystal structure of its complex with linear di-Ub (PDB ID: 3B08) are shown. **E**) Surface views of a second site on Ub recognized by the Vps23 UEV domain (Vps23 site) alone (green) and merged with the NOD1 CARD site (yellow). The Vps23 UEV domain binds both the hydrophobic pocket and this second site on Ub. Residues on Ub interacting within 3.6 Å of the UEV domain in a crystal structure of its complex with mono-Ub (PDB ID: 1UZX) are shown.

The two common binding sites on Ub are the canonical hydrophobic pocket recognized by the majority of UBDs (which is partially buried in the Ub dimer interface in the closed Lys48-linked conformation – see Figure 1, Ile 44 black spheres) and the C-terminus, which is critical in the process of conjugation by ligases and cleavage by deubiquitinating enzymes. In our structure, NOD1 binds a different surface encompassing about 500 Å^2^ that is centered on Phe4 (Figure 3A,B). Here, Thr66 is completely buried in the interface, and Lys63 and Glu64 make considerable contributions to binding NOD1 CARD. The sidechain of Glu64 on Ub, for instance, participates in six hydrogen bonds with four different residues on NOD1 CARD and also forms an edgewise intramolecular interaction with the aromatic ring of Phe4. This latter interaction is seen variably in other Ub structures (e.g. UEV domain of Vps23/TSG101:Ub (1UZX), CUE domain of Cue2:Ub (1OTR) and Lys48-linked tetra Ub (2O6V) display similar contacts, while CUE domain of Vps9p:Ub (1P3Q), mono-Ub (1UBQ) and ZnF UBP:Ub (2G45) do not), and it slightly alters the topology of the β-sheet on Ub by extending the β4/β5 loop at the expense of the fifth β-strand. In our structure, this may serve to better position Lys63 and Glu64 at the interface, as well as to orient the Phe4 aromatic sidechain for interaction with the modified Cys59 sidechain in the cleft between the second and third helices of NOD1 CARD.

Additional amphipathic residues from the first and second β-strands of Ub participate in the interactions, most notably the sidechains of Gln2, Thr14 and Glu16. Furthermore, the involvement of Lys6 extends the hydrophilic patch toward the traditional hydrophobic pocket, being in close proximity to the functionally important Leu8 residue. The remainder of the interface participates in relatively weak van der Waals interactions with NOD1 CARD. Interestingly, this surface of Ub overlaps with residues (e.g. Phe4) previously found to be functionally important for endocytosis [32], as well as with interaction sites used by other Ub-binding domains, including the UEV domain of TSG101/Vps23 [33], the NZF domain of HOIL-1L [34] and NEMO [35]; the first of these uses this patch to bind mono-Ub, while the latter two use the I44 hydrophobic patch simultaneously to bind linear poly-Ub (Figure 3C,D,E).

### Ubiquitin binds a novel site on NOD1 CARD

**Figure 4:**
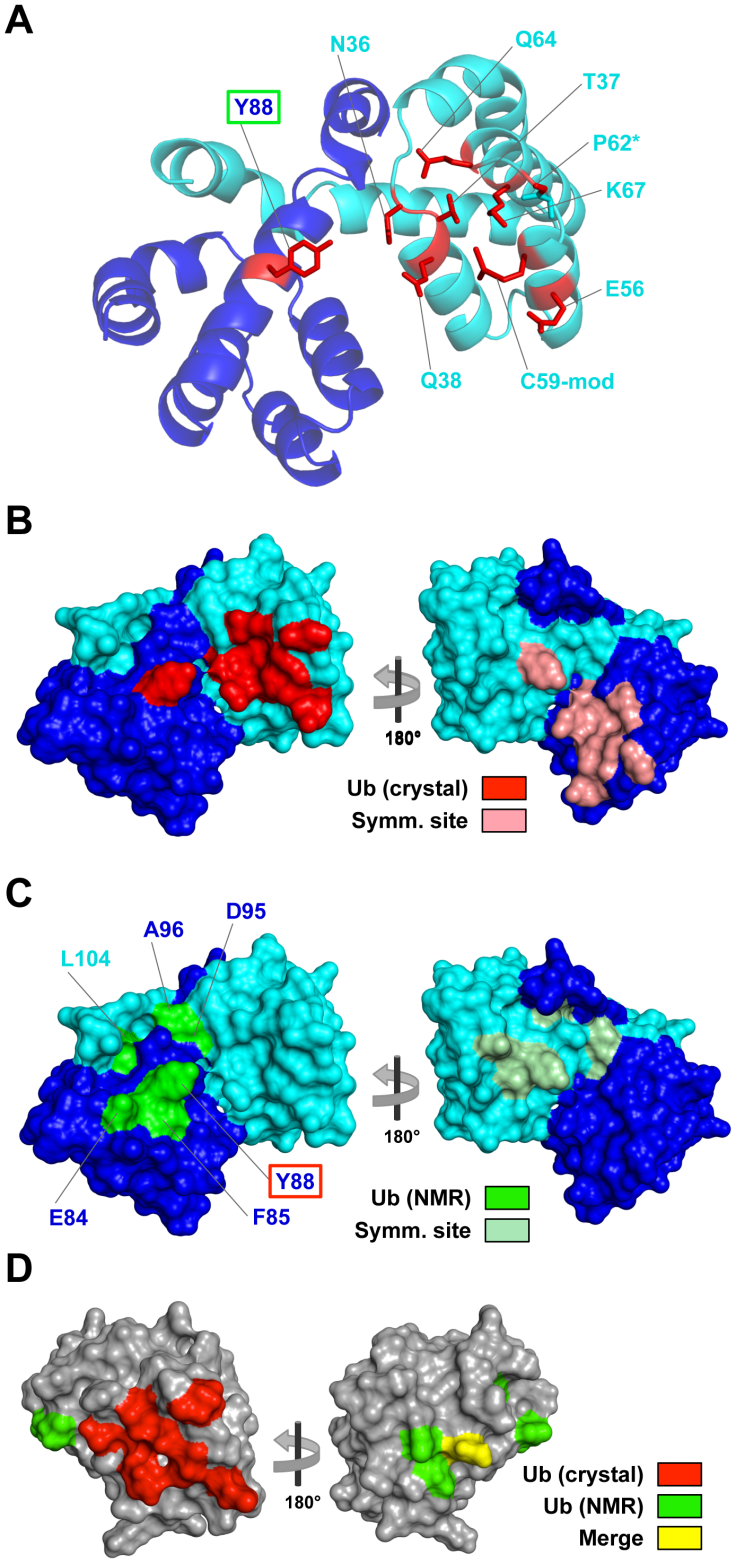
Ub interaction sites on NOD1 CARD. **A**) The NOD1 CARD dimer from our crystal structure (CARD A, cyan and CARD B, blue) is depicted in cartoon view. Residues mediating interaction with Ub (red) are shown as sticks. The asterisk on Pro62 indicates that only the main chain of this residue is involved in the interaction. The Cys59 sulfhydryl is modified with a dimethyl-As adduct (C59-mod – see figure 4). The green box around Tyr88 signifies that this residue is also involved in the previously identified NMR Ub-binding site. **B**) Surface views of the NOD1 CARD dimer showing the Ub binding site in the crystal. The majority of the binding site is on CARD A in the asymmetric unit, however Tyr88 from the CARD B subunit of the dimer is also involved in the interaction. The symmetrical binding site on the opposite side of the dimer is shown (pink) on the rotated view. **C**) Surface views of the NOD1 CARD dimer showing the Ub binding site previously identified by NMR (green and light green). This site is located primarily on helix α5, which is on a different surface of NOD1 CARD than the one in the crystal structure. In the dimer, the two sites occupy the same overall surface formed from both subunits, while in the monomer they are on different surfaces (see D). Due to helix swapping in the dimer, the Leu104 sidechain is derived from helix 6 on the opposite subunit. The red box around Tyr88 signifies that it is also involved in the Ub-binding site observed in the crystal. **D**) Surface views of the NOD1 CARD monomer (PDB ID: 2DBD) with both Ub-binding sites mapped onto it. This demonstrates that the sites occupy different surfaces on the monomer, with the implication that the full interaction observed in the crystal could not occur with the monomeric form of NOD1 CARD unless Tyr88 was not involved. This arrangement of two different binding sites on two different surfaces is similar to that seen in the structure of the HOIL-1L NZF domain bound to linear di-Ub [34].

The Ub interface on NOD1 CARD is localized primarily to an apical region of the molecule where the second, third and fourth helices converge (Figure 1, Figure 4A,B). The interaction is anchored in large part by the α1/α2 loop region and the fourth helix of NOD1 CARD, with the sidechains of Asn36, Thr37 and Gln64 hydrogen bonding with the sidechain of Glu64 from Ub. Gln64 of NOD1 CARD also hydrogen bonds with the functionally significant Lys63 sidechain of Ub. A second hub in the interface is centered on the third and fourth helices and the intervening loop region of NOD1 CARD, which bind to Thr66 of Ub. Two noteworthy contributions to the NOD1 CARD:Ub interaction arise from the third helix. First, Glu56 of NOD1 CARD is involved in a salt bridge with Lys6 of Ub, which is the only salt bridge found at the NOD1 CARD:Ub interface. Perhaps most interesting is the interaction between the sidechain of Phe4 of Ub and the chemically modified sidechain of Cys59 on NOD1 CARD (Figure 4).

Overall, the NOD1 CARD:Ub interface in our structure is small, and thus the affinity for the interaction would be expected to be weak. Indeed, in previously reported NMR solution studies of the interaction of NOD1 CARD with Ub, titrations of ^15^N-labeled NOD1 CARD with poly-Ub did not reveal an interaction surface similar to that observed in our structure, even at millimolar concentrations [14]. The chemical shift perturbations on labeled NOD1 CARD caused by poly-Ub were relatively weak, however, which necessitated rather stringent cutoffs that may have masked more minor interactions. A notable overlap in Ub-binding sites between our structure and the previous study centers on Tyr88 of NOD1 CARD, which was reported as the most perturbed residue in the presence of poly-Ub and which blocked Ub-binding when mutated. While almost the entirety of the NOD1 CARD:Ub interaction in our structure is mediated by the NOD1 CARD A and Ub C moieties in the asymmetric unit (Figure 1), the sidechain of Tyr88 from the opposing dimer subunit (NOD1 CARD B) also interacts with Ub C (Figure 4A,B,C). Its sidechain hydroxyl initiates a hydrogen bond network that interacts sequentially through the sidechains of Glu16 and Gln2 of Ub to terminate at the ε-NH2 of Gln38 on the opposite NOD1 CARD subunit. Although this interaction only buries an additional 75 Å^2^ of surface area, this represents a 15% increase over the roughly 500 Å^2^ at the primary interface.

## Discussion

Here we show the first crystal structure of the NOD1 CARD with Ub. The finding that Ub monomers crystallized as dimers similar in conformation to Lys48-linked dimers is presented here as an interesting finding mostly because it reveals a new interface between Ub and NOD1 that we did not predict from our previous studies using NMR. Importantly, this structure does not necessarily support the idea that NOD1 CARD has specificity to Lys48-linked poly-Ub. Rather, the interaction between NOD1 and Ub can happen through multiple interfaces and that particular combinations may work together to mediate binding of different poly-Ub chain topologies. It is tempting to speculate that NOD1 interactions with different poly-Ub chains could result in distinct conformations of the NOD1:Ub complex that could mediate distinct biological effects. In general, UBDs that show specificity for particular poly-Ub chains are composed of two binding-surfaces arranged so that two Ubs in a chain have simultaneous access to these sites. Often, this involves binding of one Ub via its Leu8, Ile44, V70 hydrophobic surface combined with binding to a second Ub moiety through a similar or distinct surface. Although this second surface of Ub can be quite small and have negligible affinity on its own, it can contribute substantially to the overall strength of binding in the context of a poly-Ub chain. The interaction we found in the crystal could represent such an auxiliary binding site and might provide a mechanism to explain how NOD1 CARD favors binding to Lys63 and linear poly-Ub. This model would also be consistent and complementary to our previous studies examining how the NOD1 CARD binds monomeric Ub in solution through surfaces in both the CARD and Ub that are distinct from those identified in the current crystal structure [14].

Our previous NMR studies mapped the major binding interface of NOD1 onto the canonical hydrophobic patch of Ub centered on Ile44, Val70 and Leu8, which in the current structure is used to mediate binding to another Ub molecule. Mutagenesis experiments in previous work showed that mutations of residues in NOD1 at this interface dramatically diminished binding of Ub, supporting the hypothesis that this previously determined binding mode was valid. Our previous data and the crystal structure presented here are best reconciled with Ub binding in two modes: a major mode which is required for measurable binding and which dominates in solution where monomeric NOD1 and monomeric Ub exist; and a minor mode, which was captured here in the crystal structure. These two interfaces could work together to impart better binding to poly-Ub or to guide specificity for binding poly-Ub chains linked via specific lysines.

**Figure 5:**
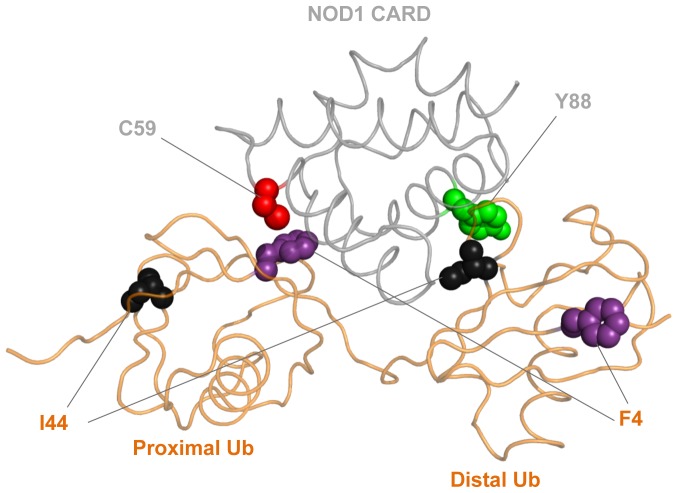
Cartoon view of a model for how monomeric NOD1 CARD could bind linear di-Ub generated by the ZDOCK server (see Materials and Methods). The sidechains of Phe4 (purple) and Ile44 (black) from Ub and Cys59 (red) and Tyr88 (green) are shown as spheres. The combination of the previously identified NMR Ub-binding site (Tyr88:Ile44) with the new Ub-binding site observed in our crystal structure (Cys59:Phe4) may explain the mechanism by which NOD1 CARD preferentially binds linear and Lys63-linked poly-Ub.

The alternative interface we discover in the crystal structure could potentially mediate di-Ub binding even for Ubs linked by chains other than Lys48, such as Lys63 and linear linked Ub chains that are known to have important signal transduction roles in innate immunity. The recent structure of the HOIL-1L NZF domain in a complex with poly-Ub is instructive with regard to our proposed model for NOD1 binding. HOIL-1L NZF binds two surfaces on Ub, one centered on the Phe4 patch and the other on the Ile44 hydrophobic pocket [34]. These two small surface patches of ∼400 Å^2^ each combine to yield a much greater affinity for poly-Ub chains when the NZF domain engages both as it “wedges” between two Ub moieties in a linear poly-Ub chain. Perhaps CARDs similarly engage two Ub surfaces, which would explain how both NOD1 CARD and RIG-I CARD [13] prefer binding to poly-Ub chains. Such a model is shown in Figure 5. Here, docking experiments were used to rationalize both the CARD:Ub interaction found in the crystal structure and the interaction earlier identified between the Glu84 and Tyr88 surface of NOD1 CARD and the canonical hydrophobic pocket of Ub (Leu8, Ile44, and Val70). These computations reveal that it is possible for the conformation of linear di-Ub observed in the HOIL-1L NZF:di-Ub structure (PDB ID: 3B08) to engage both surfaces of monomeric NOD1 CARD as it wedges between the two Ub moieties. While this model is purely speculative, it provides the basis for additional studies not only into the role of Ub in NOD1 signaling, but also in other systems where Ub is known to modulate the signaling of pathways containing various DD superfamily members.

An additional aspect of this interface is that it includes two residues of NOD1 (Asn36 and Glu56) that are highly conserved across evolution, supporting the idea that it may be used for important protein interactions such as that of Ub described here [36]. Moreover, mutation of Gln56 compromises the ability of NOD1 to activate its downstream effector kinase RIP2 [37], through a yet unknown mechanism. This interface also includes Ser65 of Ub, which was recently found to undergo phosphorylation by PINK to create a form of Ub that could act allosterically to activate the Parkin Ub ligase [38]–[40]. Thus, it may be that the binding surface on Ub described here could operate for other Ub interactions and have the capacity to be regulated.

As in any crystal structure, there exists the possibility that the unique milieu of the crystal itself produced an artifactual interaction. Although this binding interaction was not definitively observed in our prior NMR experiments, it was not definitively excluded either, given the low signal to noise of experiments performed in the range of low binding affinities expected for this interaction. Bosanac *et al* [41] reported a crystal structure of the zinc-finger domain of the protein A20 bound to three separate mono-Ubs with each site having progressively lower binding affinities. Our structure may represent even further progression to beyond the limit of detection by NMR or traditional pull-downs, where the majority of the binding events in solution represent the dominant interaction previously identified. Additionally, the exact binding mode and stoichiometry of protein complexes is not always in agreement between rigorous studies by NMR vs crystallography, as exemplified by the studies of the NEMO:linear di-Ub interaction [35], [42].

The biological relevance of this new Ub binding surface and the ability of NOD1 to bind Ub or other Ub-interacting proteins remains for future studies.

## Materials and Methods

### Protein purification

The CARD of human NOD1, from residues serine 16 to glycine 108, was produced in a pET-21a vector (Novagen). Amino acids leucine and glutamic acid were inserted between glycine 108 and the 6xHis-tag, due to the presence of an *Xho*I restriction site. The resulting plasmid was used to transform *Escherichia coli* strain BL21(DE3) (Invitrogen), which was grown at 310 K in LB medium, supplemented with ampicillin (100 µg/mL). At mid-log phase (OD_600_ = 0.5), overexpression of NOD1 CARD was induced with 1 mM isopropyl-d-thiogalactoside (Research Products International Corp.). The culture was harvested 4 hours after induction by centrifugation for 30 min at 7280 g and 277 K. The cells were resuspended in 45 mL of buffer A (50 mM sodium phosphate, 150 mM NaCl, and 5 mM imidazole, pH 7.0) per 1 L of culture, and lysed using a French press at 16,000 psi. The lysate was spun for 45 minutes at 15,000 g and 277 K to separate the soluble and insoluble fractions. The resulting supernatant was separated from the pellet and mixed with TALON metal affinity resin (BD Biosciences) pre-equilibrated in buffer A. The mixture was allowed to incubate on a rotating platform at 277 K for 2 hours, followed by addition of the slurry to a gravity flow column. The supernatant was allowed to flow through and the resin was washed with 30 batch volumes of buffer A. The NOD1 CARD protein was eluted in 50 mM sodium phosphate, 150 mM NaCl, and 500 mM imidazole, pH 7.0. Eluted protein was concentrated and further purified by gel filtration over a preparative Superdex 75 column (GE Healthcare, Piscataway, NJ) equilibrated with GF buffer (25 mM sodium phosphate, 25 mM NaCl and 5 mM DTT, pH 7.0). Two sequential rounds of gel filtration were performed to remove residual NOD1 CARD dimer and to ensure homogeneity.

Ubiquitin was purified as previously described [43], with an additional step of gel filtration over a Superdex 75 column equilibrated in GF buffer.

### Crystallization

Purified NOD1 CARD and Ub were separately concentrated to 2 mM, which was determined by absorbance at 280 nm using respective extinction coefficients of 9970 and 1280 M^−1^*cm^−1^. The separate proteins were mixed at a ratio of 1∶1 (based on prior experiments suggesting 1∶1 binding stoichiometry [14]) and incubated on ice for an hour to form complexes at a final concentration of 1 mM for each component. 2 mM complexes were created by two-fold concentration of 1 mM complexes after mixing. Complexes at concentrations less than 1 mM were made by diluting 1 mM complexes in appropriate volumes of GF buffer. Initial 96-well crystal screens utilizing the hanging drop/vapor diffusion method were set up for complexes at 2 mM or 1 mM using a TTP LabTech mosquito robot (Cambridge, MA) set to dispense 400 nL of protein solution plus 400 nL of well solution for each condition. Duplicate screens were set up and one each was left at either 277 or 291 K. Hits were obtained in PACT Suite (Qiagen) conditions #29 and #30. These contained 100 mM PCB (sodium propionate, sodium cacodylate, bis-tris propane mixed at a molar ratio of 2∶1∶2 and pH adjusted with HCl – as described by the manufacturer) and 25% PEG 1500 at pH 8.0 or 9.0, respectively. No additional DTT was added to the drops. Crystals grew overnight at both 277 and 291 K, although growth was faster at 291 K. Crystals reached a maximum size of ∼50 µM in longest dimension after two to three days. Optimization in a matrix that varied the concentration of PEG 1500, pH and protein concentration determined that the initial hit with PACT suite condition #30 (see above) was best. The crystals used for data collection were grown in 4 µL drops containing 1 mM NOD1 CARD:Ub complex and PACT Suite #30 well solution in a 1∶1 ratio.

### Data collection

Looped crystals were flash cooled in liquid N_2_. Several cryoprotectants were able to reduce ice formation, but also resulted in poorer diffraction. Ultimately, crystals used for data collection were flash frozen without additional cryoprotectant and screened for the best combination of minimal ice and maximal diffraction on a rotating anode (RU-H3R) Rigaku generator (1.54 Å) home X-ray source. Full datasets were collected on the best crystals on beamline 4.2.2 at ALS, Berkeley, California.

### Data processing, structural calculations and refinement

Diffraction datasets were processed using d*TREK (Rigaku) [44]. Phases were determined by molecular replacement with NOD1 CARD (PDB ID 2NSN) and Ub (PDB ID 1UQB) as the targets using Phaser [45]. Refinement and model building were done using Phenix [46] and Coot [47], respectively. Processing statistics are summarized in Table 1. Figures were produced using Pymol [48].

**Table 1:** X-ray data collection and refinement statistics.

**Data Collection**	
Wavelength (Å)	1.000
Space group	P4_1_2_1_2
Cell dimensions (Å)	*a* = *b* = 61.69, *c* = 86.87
Cell Angles (°)	α = β = γ = 90
Resolution range (Å)	38.98 – 2.90 (3.00 – 2.90)
Total reflections	54090
Unique reflections	4048 (395)
Completeness (%)	99.88 (100.0)
Multiplicity	13.35 (13.92)
*R_merge_*	0.05 (0.21)
I/σ (I)	28.40 (11.40)
**Refinement**	
Resolution range (Å)	38.98 – 2.90
Number of reflections in working set	3642
Number of reflections in test set	406
*R_work_*	0.226
*R_free_*	0.270
R.M.S.D.	
Bond lengths (Å)	0.003
Bond angles (°)	0.704
No. protein atoms	
Chain *A*	751
Chain *B*	582
No. ligand atoms	5
Average *B* factor (Å^2^)	57.40
Ramachandran favored (%)	98
Ramachandran outliers (%)	1.2

Values for the highest resolution shell are given in parentheses.

### Accession numbers

The structure for the NOD1 CARD:Ub complex was deposited in the Protein Data Bank under ID code 4JQW.

### Docking calculations

To model the proposed similarity of the HOIL-1L NZF interaction with linear di-Ub to how NOD1 CARD might also bind linear poly-Ub, NOD1 CARD monomer (PDB ID: 2DBD) was paired with linear di-Ub from the HOIL-1L NZF:di-Ub crystal structure (PDB ID: 3B08) to predict possible complexes using the ZDOCK server [49], [50]. Results were filtered by the criteria that Tyr88 of NOD1 CARD interacted with Ile44 of the distal Ub moiety (NMR site) and Cys59 of NOD1 CARD interacted with Phe4 of the proximal Ub moiety (crystal site).

## References

[1] Girardin S, Boneca I, Carneiro L, Antignac A, Jehanno M, et al (2003) Nod1 detects a unique muropeptide from gram-negative bacterial peptidoglycan. Science 300: 1584–1587. 12791997 10.1126/science.1084677

[2] Travassos LH, Carneiro LAM, Ramjeet M, Hussey S, Kim Y-G, et al (2010) Nod1 and Nod2 direct autophagy by recruiting ATG16L1 to the plasma membrane at the site of bacterial entry. Nat Immunol 11: 55–62 10.1038/ni.1823 19898471

[3] Viala J, Chaput C, Boneca I, Cardona A, Girardin S, et al (2004) Nod1 responds to peptidoglycan delivered by the Helicobacter pylori cag pathogenicity island. Nat Immunol 5: 1166–1174. 15489856 10.1038/ni1131

[4] Clarke TB, Davis KM, Lysenko ES, Zhou AY, Yu Y, et al (2010) Recognition of peptidoglycan from the microbiota by Nod1 enhances systemic innate immunity. Nat Med 16: 228–231 10.1038/nm.2087 20081863 PMC4497535

[5] Bouskra D, Brézillon C, Bérard M, Werts C, Varona R, et al (2008) Lymphoid tissue genesis induced by commensals through NOD1 regulates intestinal homeostasis. Nature 456: 507–510 10.1038/nature07450 18987631

[6] Schertzer JD, Tamrakar AK, Magalhães JG, Pereira S, Bilan PJ, et al (2011) NOD1 Activators Link Innate Immunity to Insulin Resistance. Diabetes 10.2337/db11-0004 PMC3161332 21715553

[7] Hysi P, Kabesch M, Moffatt MF, Schedel M, Carr D, et al (2005) NOD1 variation, immunoglobulin E and asthma. Hum Mol Genet 14: 935–941 10.1093/hmg/ddi087 15718249

[8] Cardenas I, Mulla MJ, Myrtolli K, Sfakianaki AK, Norwitz ER, et al (2011) Nod1 Activation by Bacterial iE-DAP Induces Maternal-Fetal Inflammation and Preterm Labor. J Immunol 187: 980–986 10.4049/jimmunol.1100578 21677137

[9] Inohara N, Koseki T, del Peso L, Hu Y, Yee C, et al (1999) Nod1, an Apaf-1-like activator of caspase-9 and nuclear factor-κB. Journal of Biological Chemistry 274: 14560–14567. 10329646 10.1074/jbc.274.21.14560

[10] Inohara N, Koseki T, Lin J, del Peso L, Lucas P, et al (2000) An induced proximity model for NF-kappa B activation in the Nod1/RICK and RIP signaling pathways. J Biol Chem 275: 27823–27831. 10880512 10.1074/jbc.M003415200

[11] Park H, Lo Y, Lin S, Wang L, Yang J, et al (2007) The death domain superfamily in intracellular signaling of apoptosis and inflammation. Annu Rev Immunol 25: 561–86. 17201679 10.1146/annurev.immunol.25.022106.141656 PMC2904440

[12] Jiang X, Kinch LN, Brautigam CA, Chen X, Du F, et al (2012) Ubiquitin-Induced Oligomerization of the RNA Sensors RIG-I and MDA5 Activates Antiviral Innate Immune Response. Immunity 36: 959–973 10.1016/j.immuni.2012.03.022 22705106 PMC3412146

[13] Zeng W, Sun L, Jiang X, Chen X, Hou F, et al (2010) Reconstitution of the RIG-I pathway reveals a signaling role of unanchored polyubiquitin chains in innate immunity. Cell 141: 315–330 10.1016/j.cell.2010.03.029 20403326 PMC2919214

[14] Ver Heul AM, Fowler CA, Ramaswamy S, Piper RC (2013) Ubiquitin regulates caspase recruitment domain-mediated signaling by nucleotide-binding oligomerization domain-containing proteins NOD1 and NOD2. Journal of Biological Chemistry 288: 6890–6902 10.1074/jbc.M112.413781 23300079 PMC3591598

[15] Palombella VJ, Rando OJ, Goldberg AL, Maniatis T (1994) The ubiquitin-proteasome pathway is required for processing the NF-kappa B1 precursor protein and the activation of NF-kappa B. Cell 78: 773–785. 8087845 10.1016/s0092-8674(94)90482-0

[16] Liu S, Chen ZJ (2011) Expanding role of ubiquitination in NF-κB signaling. Cell Res 21: 6–21 10.1038/cr.2010.170 21135871 PMC3193409

[17] Ikeda F, Dikic I (2008) Atypical ubiquitin chains: new molecular signals. “Protein Modifications: Beyond the Usual Suspects” review series. EMBO Rep 9: 536–542 10.1038/embor.2008.93 18516089 PMC2427391

[18] Malynn BA, Ma A (2010) Ubiquitin makes its mark on immune regulation. Immunity 33: 843–852 10.1016/j.immuni.2010.12.007 21168777 PMC3030984

[19] Bhoj VG, Chen ZJ (2009) Ubiquitylation in innate and adaptive immunity. Nature 458: 430–437 10.1038/nature07959 19325622

[20] Xia Z-P, Sun L, Chen X, Pineda G, Jiang X, et al (2009) Direct activation of protein kinases by unanchored polyubiquitin chains. Nature 461: 114–119 10.1038/nature08247 19675569 PMC2747300

[21] Srimathi T, Robbins SL, Dubas RL, Hasegawa M, Inohara N, et al (2008) Monomer/dimer transition of the caspase-recruitment domain of human Nod1. Biochemistry 47: 1319–1325 10.1021/bi7016602 18186648

[22] Coussens NP, Mowers JC, McDonald C, Nuñez G, Ramaswamy S (2007) Crystal structure of the Nod1 caspase activation and recruitment domain. Biochem Biophys Res Commun 353: 1–5 10.1016/j.bbrc.2006.11.122 17173864 PMC1821002

[23] Eddins MJ, Varadan R, Fushman D, Pickart CM, Wolberger C (2007) Crystal structure and solution NMR studies of Lys48-linked tetraubiquitin at neutral pH. J Mol Biol 367: 204–211 10.1016/j.jmb.2006.12.065 17240395

[24] Li X, Han Y, Pan XM (2001) Cysteine-25 of adenylate kinase reacts with dithiothreitol to form an adduct upon aging of the enzyme. FEBS Lett 507: 169–173. 11684092 10.1016/s0014-5793(01)02954-4

[25] Zhu X (2002) Observation of an Arsenic Adduct in an Acetyl Esterase Crystal Structure. Journal of Biological Chemistry 278: 2008–2014 10.1074/jbc.M210103200 12421810

[26] Chin K-H, Tsai Y-D, Chan N-L, Huang K-F, Wang AHJ, et al (2007) The crystal structure of XC1258 from Xanthomonas campestris: A putative procaryotic Nit protein with an arsenic adduct in the active site. Proteins 69: 665–671 10.1002/prot.21501 17640068

[27] Liu X, Zhang H, Wang X-J, Li L-F, Su X-D (2011) Get phases from arsenic anomalous scattering: de novo SAD phasing of two protein structures crystallized in cacodylate buffer. PLoS ONE 6: e24227 10.1371/journal.pone.0024227 21912678 PMC3166297

[28] Tsao DHH, Maki AH (1991) Optically detected magnetic resonance study of the interaction of an arsenic(III) derivative of cacodylic acid with EcoRI methyltransferase. Biochemistry 30: 4565–4572 10.1021/bi00232a029 2021649

[29] Maignan S, Guilloteau JP, Zhou-Liu Q, Clément-Mella C, Mikol V (1998) Crystal structures of the catalytic domain of HIV-1 integrase free and complexed with its metal cofactor: high level of similarity of the active site with other viral integrases. J Mol Biol 282: 359–368 10.1006/jmbi.1998.2002 9735293

[30] Varadan R, Walker O, Pickart C, Fushman D (2002) Structural properties of polyubiquitin chains in solution. J Mol Biol 324: 637–647. 12460567 10.1016/s0022-2836(02)01198-1

[31] Qureshi IA, Ferron F, Seh C, Cheung P, Lescar J (2009) Crystallographic structure of ubiquitin in complex with cadmium ions. BMC research notes 2: 251 10.1186/1756-0500-2-251 20003470 PMC2804574

[32] Sloper-Mould KE, Jemc JC, Pickart CM, Hicke L (2001) Distinct functional surface regions on ubiquitin. J Biol Chem 276: 30483–30489 10.1074/jbc.M103248200 11399765

[33] Teo H, Veprintsev DB, Williams RL (2004) Structural insights into endosomal sorting complex required for transport (ESCRT-I) recognition of ubiquitinated proteins. J Biol Chem 279: 28689–28696 10.1074/jbc.M400023200 15044434

[34] Sato Y, Fujita H, Yoshikawa A, Yamashita M, Yamagata A, et al (2011) Specific recognition of linear ubiquitin chains by the Npl4 zinc finger (NZF) domain of the HOIL-1L subunit of the linear ubiquitin chain assembly complex. Proc Natl Acad Sci USA 108: 20520–20525 10.1073/pnas.1109088108 22139374 PMC3251058

[35] Lo Y-C, Lin S-C, Rospigliosi CC, Conze DB, Wu C-J, et al (2009) Structural basis for recognition of diubiquitins by NEMO. Mol Cell 33: 602–615 10.1016/j.molcel.2009.01.012 19185524 PMC2749619

[36] Boyle JP, Mayle S, Parkhouse R, Monie TP (2013) Comparative Genomic and Sequence Analysis Provides Insight into the Molecular Functionality of NOD1 and NOD2. Front Immunol 4: 317 10.3389/fimmu.2013.00317 24109482 PMC3791470

[37] Manon F, Favier A, Favier A, Núñez G, Núñez G, et al (2007) Solution structure of NOD1 CARD and mutational analysis of its interaction with the CARD of downstream kinase RICK. J Mol Biol 365: 160–174 10.1016/j.jmb.2006.09.067 17054981

[38] Koyano F, Okatsu K, Kosako H, Tamura Y, Go E, et al (2014) Ubiquitin is phosphorylated by PINK1 to activate parkin. Nature 510: 162–166 10.1038/nature13392 24784582

[39] Kazlauskaite A, Kondapalli C, Gourlay R, Campbell DG, Ritorto MS, et al (2014) Parkin is activated by PINK1-dependent phosphorylation of ubiquitin at Ser65. Biochem J 460: 127–139 10.1042/BJ20140334 24660806 PMC4000136

[40] Kane LA, Lazarou M, Fogel AI, Li Y, Yamano K, et al (2014) PINK1 phosphorylates ubiquitin to activate Parkin E3 ubiquitin ligase activity. The Journal of Cell Biology 205: 143–153 10.1083/jcb.201402104 24751536 PMC4003245

[41] Bosanac I, Wertz IE, Pan B, Yu C, Kusam S, et al (2010) Ubiquitin binding to A20 ZnF4 is required for modulation of NF-κB signaling. Mol Cell 40: 548–557 10.1016/j.molcel.2010.10.009 21095585

[42] Rahighi S, Ikeda F, Kawasaki M, Akutsu M, Suzuki N, et al (2009) Specific recognition of linear ubiquitin chains by NEMO is important for NF-kappaB activation. Cell 136: 1098–1109 10.1016/j.cell.2009.03.007 19303852

[43] Pashkova N, Gakhar L, Winistorfer SC, Yu L, Ramaswamy S, et al (2010) WD40 repeat propellers define a ubiquitin-binding domain that regulates turnover of F box proteins. Mol Cell 40: 433–443 10.1016/j.molcel.2010.10.018 21070969 PMC3266742

[44] Pflugrath JW (1999) The finer things in X-ray diffraction data collection. Acta Crystallogr D Biol Crystallogr 55: 1718–1725. 10531521 10.1107/s090744499900935x

[45] McCoy AJ, Grosse-Kunstleve RW, Adams PD, Winn MD, Storoni LC, et al (2007) Phaser crystallographic software. J Appl Crystallogr 40: 658–674 10.1107/S0021889807021206 19461840 PMC2483472

[46] Adams PD, Afonine PV, Bunkóczi G, Chen VB, Davis IW, et al (2010) PHENIX: a comprehensive Python-based system for macromolecular structure solution. Acta Crystallogr D Biol Crystallogr 66: 213–221 10.1107/S0907444909052925 20124702 PMC2815670

[47] Emsley P, Cowtan K (2004) Coot: model-building tools for molecular graphics. Acta Crystallogr D Biol Crystallogr 60: 2126–2132 10.1107/S0907444904019158 15572765

[48] Schrödinger, LLC (n.d.) The PyMOL Molecular Graphics System, Version 1.5.0.4. Schrödinger, LLC.

[49] Chen R, Li L, Weng Z (2003) ZDOCK: an initial-stage protein-docking algorithm. Proteins 52: 80–87 10.1002/prot.10389 12784371

[50] Pierce BG, Hourai Y, Weng Z (2011) Accelerating protein docking in ZDOCK using an advanced 3D convolution library. PLoS ONE 6: e24657 10.1371/journal.pone.0024657 21949741 PMC3176283

